# Consequences of the Lack of CD73 and Prostatic Acid Phosphatase in the Lymphoid Organs

**DOI:** 10.1155/2014/485743

**Published:** 2014-08-24

**Authors:** Gennady G. Yegutkin, Kaisa Auvinen, Marika Karikoski, Pia Rantakari, Heidi Gerke, Kati Elima, Mikael Maksimow, Ileana B. Quintero, Pirkko Vihko, Marko Salmi, Sirpa Jalkanen

**Affiliations:** ^1^MediCity Research Laboratory, University of Turku, Tykistökatu 6 A, 20520 Turku, Finland; ^2^Department of Medical Microbiology and Immunology, University of Turku, 20014 Turku, Finland; ^3^National Institute for Health and Welfare, 20520 Turku, Finland; ^4^Department of Physiology and Turku Center for Disease Modeling, Institute of Biomedicine, University of Turku, 20014 Turku, Finland; ^5^Department of Medical Biochemistry and Genetics, University of Turku, 20014 Turku, Finland; ^6^Department of Clinical Chemistry, and Helsinki University Hospital Laboratory, University of Helsinki, 00014 Helsinki, Finland

## Abstract

CD73, ecto-5′-nucleotidase, is the key enzyme catalyzing the conversion of extracellular AMP to adenosine that controls vascular permeability and immunosuppression. Also prostatic acid phosphatase (PAP) possesses ecto-5′-nucleotidase/AMPase activity and is present in leukocytes. However, its role related to immune system is unknown. Therefore, we analyzed enzymatic activities and leukocyte subtypes of CD73 and PAP knockouts and generated CD73/PAP double knockout mice to elucidate the contribution of CD73 and PAP to immunological parameters. Enzymatic assays confirmed the ability of recombinant human PAP to hydrolyze [^3^H]AMP, although at much lower rate than human CD73. Nevertheless, 5′-nucleotidase/AMPase activity in splenocytes and lymphocytes from PAP^−/−^ mice tended to be lower than in wild-type controls, suggesting potential contribution of PAP, along with CD73, into lymphoid AMP metabolism ex vivo. Single knockouts had decreased number of CD4^+^/CD25^+^/FoxP_3_^+^ regulatory T cells in thymus and CD73/PAP double knockouts exhibited reduced percentages of CD4^+^ cells in spleen, regulatory T cells in lymph nodes and thymus, and CD4^+^ and CD8^+^ cells in blood. These findings suggest that PAP has a synergistic role together with CD73 in the immune system by contributing to the balance of leukocyte subpopulations and especially to the number of regulatory T cells in lymph nodes and thymus.

## 1. Introduction

Balance between extracellular ATP, ADP, AMP, and adenosine controls the inflammatory status of the microenvironment, because ATP is proinflammatory while adenosine is highly anti-inflammatory. CD73 ectonucleotidase is a key enzyme in the purinergic signaling cascade as it dephosphorylates AMP and produces adenosine. Both endothelial cells and certain subsets of leukocytes express CD73. On endothelium [1–4] CD73-dependent adenosine production controls vascular permeability as clearly demonstrated by leaky vasculature and aggravated inflammations in CD73 deficient mice [5–8]. Regulatory T cells (Tregs) express CD73 and exert their immunosuppressive effects via adenosine. On the other hand, adenosine promotes Treg expansion and triggers A_2A_ adenosine receptor on effector T cells inducing NF-kB-mediated suppression of cytokine production and other anti-inflammatory effects [9, 10].

Human prostate expresses high amounts of PAP and the levels of the enzyme activity in serum has been connected to prostate cancer already decades ago [11], and in the recent years PAP has become the target for prostate cancer immunotherapy [12]. Indeed, there are two isoforms of prostatic acid phosphatase, a secretory (sPAP) and a type 1 transmembrane (TMPAP) isoform, which are splice variants encoded by the same gene (*ACPP*) [13]. PAP presents ecto-5′-nucleotidase activity, and subsequent studies have unambiguously demonstrated its wide expression beyond the prostate including leukocytes [14], and the TMPAP isoform has been found in various tissues, including spleen, thymus, and neurons [13, 15]. Besides substrates such as *β*-glycerophosphate, lysophosphatidic acid, and phosphoamino acids, PAP can also catalyze AMP [16] and, in fact, PAP-mediated adenosine formation has been shown to be an important regulator of pain by inhibiting nociceptive neurotransmission [17–19]. Thus, the studies regarding PAP have concentrated on its function in the prostate and the neural system and, unlike CD73, its role in the immune system has remained unknown. In this work, we analyzed the nucleotidase activities of purified hPAP in comparison to recombinant CD73 in vitro and, further, ex vivo by using lymphoid cell suspensions isolated from wild type, CD73 and PAP knockout, and CD73/PAP double knockout mice, as well as mouse lymph nodes and human tonsils cryosections as enzyme sources. Moreover, we show the consequences of PAP deficiency alone and combined with the absence of CD73 to the composition of immune cells in main lymphoid organs. To the best of our knowledge, this is the first demonstration of a role for PAP in the immune system.

## 2. Materials and Methods

### 2.1. Mice

*Pa* *p* ^−/−^ (mPAP KO) and *Nt*5*e* ^−/−^ (CD73 KO) were created by targeted gene disruption as previously described [20, 21]. Mice were backcrossed to C57BL/6J mice for >10 generations. dKO (mPAP KO/CD73 KO) mice were generated by breeding backcrossed *Nt*5*e* ^−/−^ and *Pap* ^−/−^ mice and the double mutant mice were selected by PCR-based genotyping. They were analyzed alongside age-matched C57BL/6J wild type mice as controls. Two- to 4-month-old mice were used. Mice were raised under a 12 : 12 light : dark cycle and used during the light phase. The mice were maintained in a specific pathogen-free stage at Central Animal Laboratory at the University of Turku, complying with international guidelines on the care and use of laboratory animals and performed in compliance with the 3Rs principle. All animal experiments were approved by the Finnish Animal Ethics Committee, project license number 3791/04.10.03/2011.

### 2.2. Purification of Human CD73 and PAP

Recombinant human CD73 was purified from transfected CHO cells. The harvest was purified using immobilized N6-(6-amino)hexyl-AMP (Jena Bioscience Gmbh, Jena, Germany). The bound rhCD73 was eluted using 0.25 M AMP in 50 mM Tris-HCl, 0.1 M NaCl, and pH 7.4. After the purification, the buffer was changed to 20 mM Tris-HCl, pH 8.0, using Amicon Ultra 30 device and the final concentration of the CD73 was measured using specific in-house ELISA.

hPAP was purified from human seminal plasma as described previously [22, 23]. Enzyme specific activity for the dephosphorylation of p-nitrophenylphosphate at pH 4.8 (50 mM Na citrate buffer) was 5500 *μ*mol/min∗mg.

### 2.3. Cell Isolation, Flow Cytometric Analyses, and Immunohistochemistry

Single cell suspensions were prepared from minced pieces of peripheral lymph nodes (pooled axial and inguinal nodes), thymus, and spleen by mechanical teasing through a metal meshwork and bone marrow cells by flushing the femurs. Blood was collected by cardiac puncture. Erythrocytes were removed from spleen and blood by hypotonic lysis. Leukocyte phenotyping was done using mAbs against CD4 (clone RM4-5, Alexa Fluor 647 conjugated), CD8 (clone 53-6.7, PerCP-Cy5.5 conjugated), and anti-B220 (clone RA3-6B2, Pacific blue conjugated), all obtained from BD Biosciences. Regulatory T cells were identified using mouse regulatory T cell staining kit (eBioscience; CD4-FITC, CD25-allophycocyanin, and FoxP3-PE). Cells were analyzed using LSRII using BDFACS-Diva software. Representative dot blots and histograms were made with FlowJo software. Frozen sections of thymuses were also stained for CD8, CD4, and CD25.

### 2.4. Thin-Layer Chromatographic (TLC) Analysis of Enzymatic Activities

Purinergic activities of isolated single cells from bone marrow, spleen, and peripheral lymph nodes were determined by using [2,8-^3^H]ATP, [2,8-^3^H]ADP (Perkin Elmer), [2-^3^H]AMP (Quotient Bioresearch, GE Healthcare, Rushden, UK), and [2-^3^H]adenosine (Amersham, Little Chalfont, UK) as described previously [24]. Briefly, the single cell suspensions (5–10 × 10^4^ cells) were incubated for 45–60 min at 37°C in a final volume of 80 *μ*L RPMI-1640 in neutral pH supplemented with 4 mmol/L *β*-glycerophosphate and the following tracer substrates: 500 *μ*M [^3^H]ATP (ATPase), 500 *μ*M [^3^H]ADP (ADPase), 300 *μ*M [^3^H]AMP (ecto-5′-nucleotidase), 300 *μ*M [^3^H]adenosine (adenosine deaminase), and 400 *μ*M [^3^H]AMP plus 600 *μ*M *γ*-phosphate-donating ATP (adenylate kinase). Likewise, AMP-hydrolyzing activity of purified human enzymes was determined by incubating PAP (1 *μ*g of protein) or CD73 (0.5 ng) with 40 *μ*M [^3^H]AMP in the absence and presence of *β*-glycerophosphate (4 mmol/L) or selective ecto-5′-nucleotidase/CD73 inhibitor *α*,*β*-methylene-ADP (AMPCP; 5 *μ*M). Mixture aliquots were applied onto Alugram G/UV_254_ sheets (Macherey-Nagel) and ^3^H-labeled substrates and their metabolites were separated by TLC and quantified by scintillation *β*-counting.

### 2.5. Enzyme Histochemistry

Localization of ecto-5′-nucleotidase/AMPase and other nucleotidase activities was determined in murine lymph node and also human tonsil cryosections by using a modification of the lead nitrate [Pb(NO_3_)_2_] method, as described previously [25, 26]. Tissue sections were also stained with hematoxylin and eosin. Slides were mounted with Aquatex (Merck) and images were acquired using an Olympus BX60 microscope.

### 2.6. Statistical Analyses

The results are presented as mean ± SEM, unless otherwise stated. Statistical analyses were done using two-sided Student's *t*-test and values of *P* < 0.05 were considered significant.

## 3. Results and Discussion

### 3.1. Activity of CD73 Dominates over PAP in AMP Hydrolysis Both In Vitro and In Vivo

**Figure 1:**
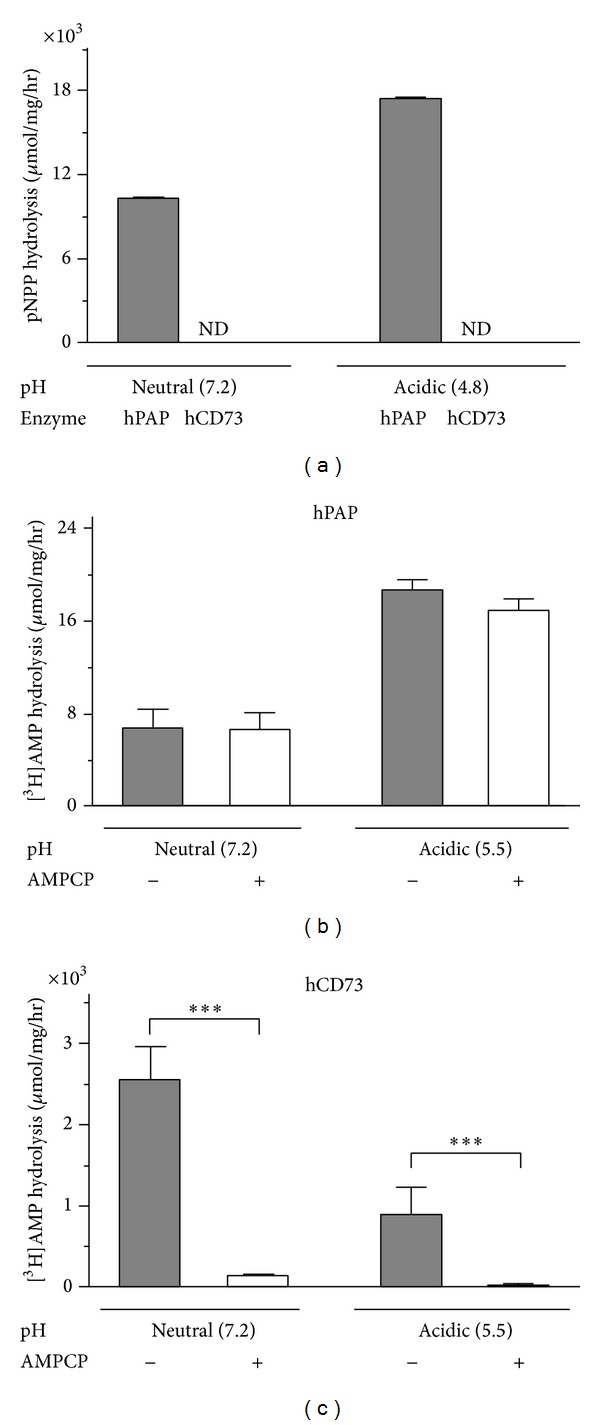
Nucleotidase activities of purified human PAP and recombinant human CD73. (a) The activities of hPAP and hCD73 were determined spectrophotometrically at neutral or acidic pH by using artificial substrate p-nitrophenylphosphate (pNPP).* ND*: nondetectable. Radio-TLC analysis of nucleotidase activities was also performed by incubating purified hPAP (b) and hCD73 (c) with 40 *μ*M [^3^H]AMP at different pH without and with 5 *μ*M AMPCP, as indicated. Enzymatic activities are expressed as *μ*moles of pNPP or [^3^H]AMP hydrolyzed per hour by one milligram of enzyme protein. _ _*P < 0.05.

As PAP has been demonstrated to contribute, along with a predominant role of CD73, to the conversion of extracellular AMP into adenosine [16, 17], we first compared the activities of purified hPAP and purified CD73. Measurement of hPAP activity revealed efficient hydrolysis of artificial substrate pNPP by the purified enzyme (Figure 1(a)) and further demonstrated its ability to metabolize [^3^H]AMP (Figure 1(b)), with highest catalytic activities being detected at acidic pH. Interestingly, AMP-hydrolyzing capability of hPAP was ~400- and ~50-fold less in comparison with purified CD73 when assayed at pH 7.2 and 5.5, respectively, and, in addition, it was not inhibited by selective ecto-5′-nucleotidase inhibitor AMPCP (Figure 1). Noteworthy, PAP could also potentially hydrolyze *β*-glycerophosphate as an alternative phosphorylated substrate [16]. However, no differences in the rates of [^3^H]AMP hydrolysis by purified hPAP or hCD73 were detected in our TLC assays performed in the absence and presence of 4 mM *β*-glycerophosphate (data not shown).

**Figure 2:**
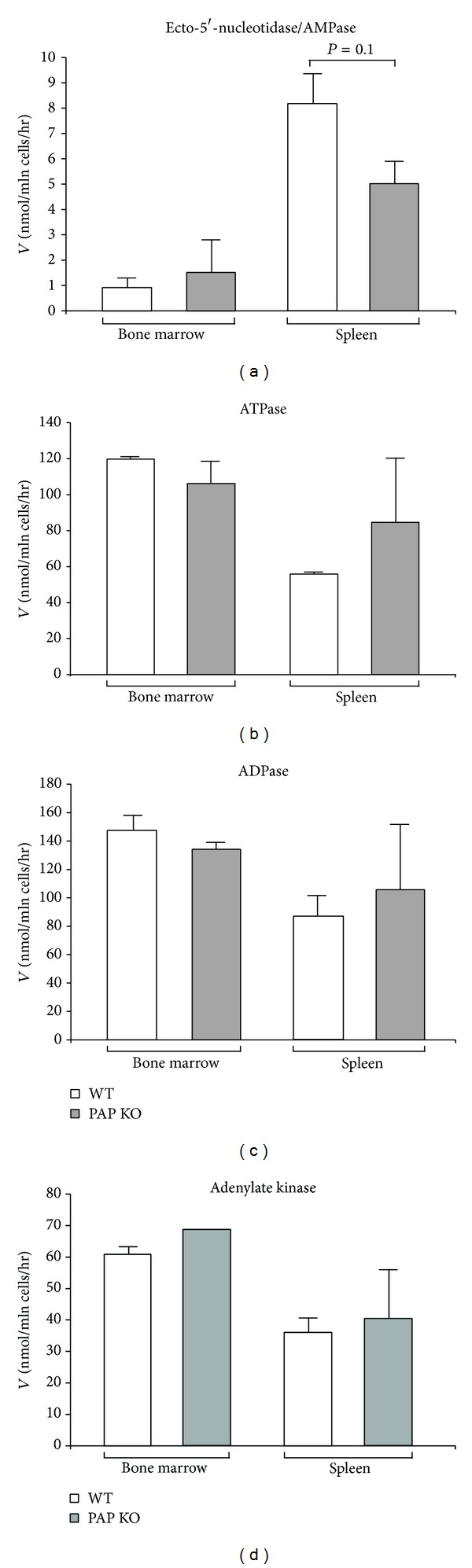
PAP deficient mice do not have significant alterations in extracellular nucleotide metabolism. The activities of ecto-5′-nucleotidase/AMPase (a), ATPase (b), ADPase (c), and adenylate kinase (d) were determined radiochemically in bone marrow and spleen leukocytes isolated from wild type and PAP knockout mice (mean ± SEM; *n* = 3–5).

To further evaluate the potential role of PAP in lymphoid nucleotide homeostasis, we measured the activities of ecto-5′-nucleotidase (Figure 2(a)) and other related purinergic enzymes, ATPase (Figure 2(b)), ADPase (Figure 2(c)), and adenylate kinase (Figure 2(d)), in lymphocytes isolated from spleen and bone marrow of PAP knockout and wild type mice. No statistical differences were found in these activities, although there was a tendency towards reduced ecto-5′-nucleotidase activity in the spleen of the PAP knockout mice. Next, we concentrated on lymphocytes isolated from peripheral lymph nodes, which drain the periphery and are the primary sites for immune response against antigens arriving from the periphery via afferent lymphatics, and analyzed the enzymatic activities in wild type, CD73, PAP, and CD73/PAP double knockouts. The double knockouts generated for this work did not show any obvious phenotype, when housed in specific pathogen-free conditions.

**Figure 3:**
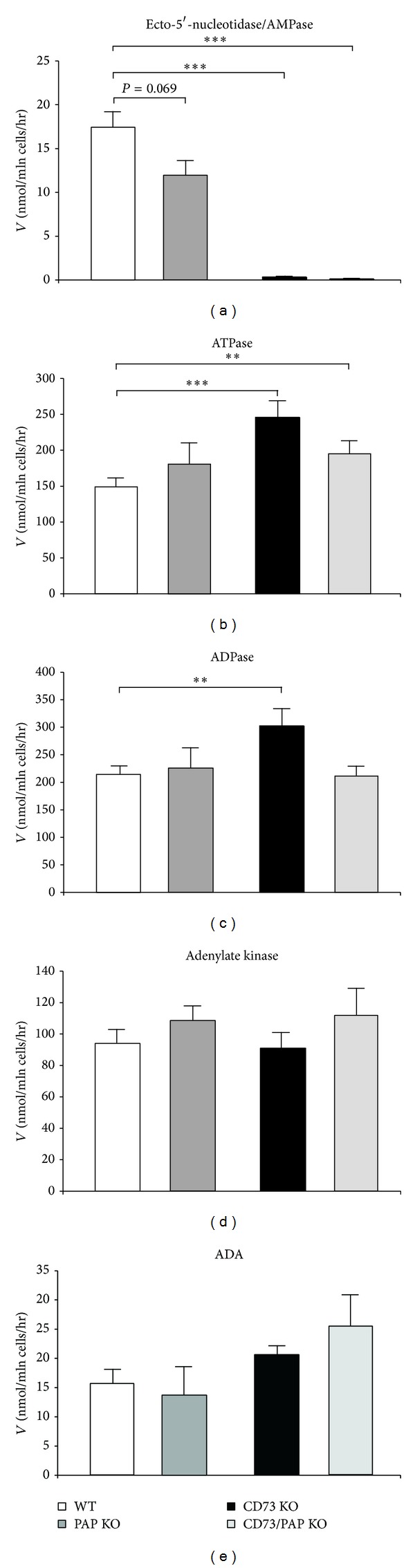
Purine-converting activities in CD73 and PAP deficient mice. The activities of ecto-5′-nucleotidase/AMPase (a), ATPase (b), ADPase (c), adenylate kinase (d), and adenosine deaminase (e) were determined by TLC in lymph node lymphocytes from wild type mice (*n* = 14), CD73 single (*n* = 7), PAP single (*n* = 9), and CD73/PAP double knockouts (*n* = 7). Enzymatic activities were expressed as nanomoles of ^3^H-substrate metabolized per hour by 10^6^ cells. _ _**P ≤ 0.01, _ _***P ≤ 0.001.

As expected, practically no AMPase activity was detected in the lymphocytes isolated from CD73 knockout mice, thus confirming that lymphoid ecto-5′-nucleotidase/CD73 indeed represents the major enzyme responsible for conversion of extracellular AMP into adenosine (Figure 3(a)). Nonetheless, as in the case of spleen studies (see Figure 2(a)), a clear trend for diminished [^3^H]AMP hydrolysis was observed in PAP^−/−^ lymphocytes (Figure 3(a)). These data suggest that PAP may contribute, along with CD73, to lymphoid AMP metabolism, presumably in certain synergistic fashion. Consistent with our previous observations [27], we also found significantly increased ATPase and ADPase activities in lymphocytes from CD73 deficient animals, with the latter nucleotidase activity remaining significantly upregulated also in CD73/PAP double knockout mice. No significant shifts in other purine-converting enzymes, adenosine deaminase and adenylate kinase, were detected among the studied genotypes (Figure 3).

### 3.2. PAP and CD73 Are Differently Expressed in Mouse and Human Lymphoid Tissues and Contribute to Nucleotide Metabolism

**Figure 4:**
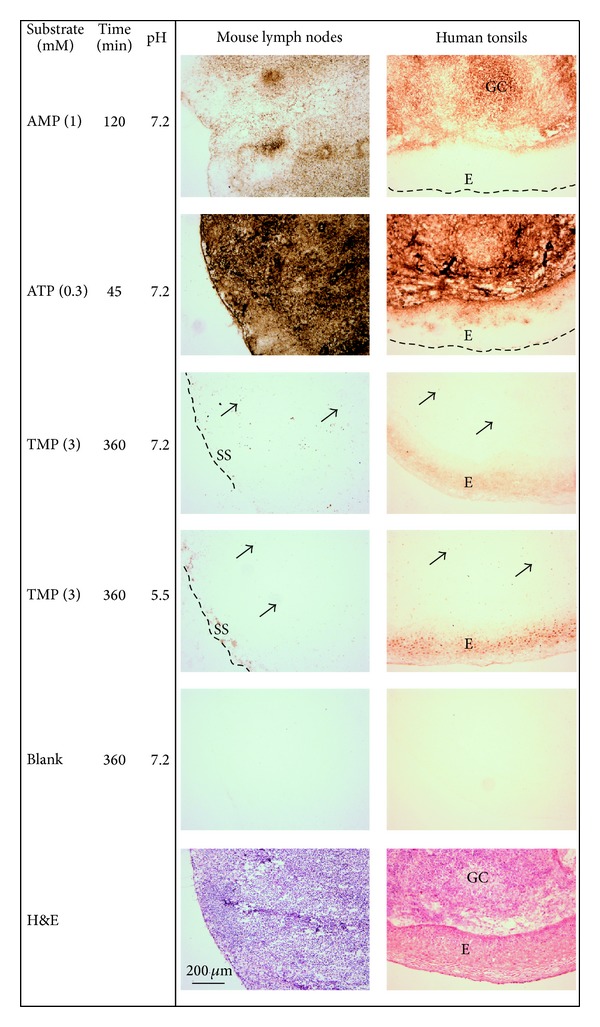
Distribution of nucleotidase activities in mouse lymph nodes and human tonsils. Enzyme histochemical staining was performed by incubating tissue sections at different pH for the indicated time without (Blank) and with different nucleotide substrates. Tissue sections were also stained with hematoxylin and eosin (H&E). The capsules are indicated by dashed lines, the arrows point at some positive scattered cells, GC: germinal center, SS: subcapsular sinus, and E: epithelium. Original magnification: ×100. Scale bar: 200 *μ*m.

The distribution of different nucleotidases in the lymphoid tissues was then evaluated in situ by a lead precipitation method. Short-time incubation of mouse lymph node and human tonsil cryosections with different nucleotide substrates revealed the presence of high AMPase and especially ATPase activities (Figure 4). The employment of TMP as a substrate and increasing the incubation time also allowed us to detect relatively moderate but clearly visible staining, which was specifically restricted to the subcapsular area of mouse lymph nodes, the epithelial layer of human tonsils, and also individual lymphoid cells scattered throughout the lymphoid tissues (Figure 4). Strikingly, the most intense TMPase staining was observed at acidic pH, suggesting the predominant contribution of PAP enzyme to the measured nucleotidase activity. Noteworthy, it has been proposed recently that selective expression of tissue-nonspecific alkaline phosphatase and another pH-dependent enzyme tartrate-resistant acid phosphatase (TRAP) in corresponding alkaline and acidic lacunas of bone might function as specific pH sensors directionally regulating nucleotide receptor-mediated osteoclast function and bone resorption [28]. It is also pertinent to mention that, under certain circumstances, like ischemia, intracellular pH is decreased to ~6.4, accompanied by marked inhibition of 5′-nucleotidase activity and changes in purine homeostasis in the heart [29]. It may be reasonably speculated that different localization of CD73 and PAP and their inverse pH dependence provide efficient means for tuned regulation of purine homeostasis in lymphoid tissues during inflammation, hypoxia, and other (patho)physiological states. Further studies are required to validate this hypothesis.

### 3.3. PAP Deficiency Has Only Minor Effect on Lymphocyte Populations

**Figure 5:**
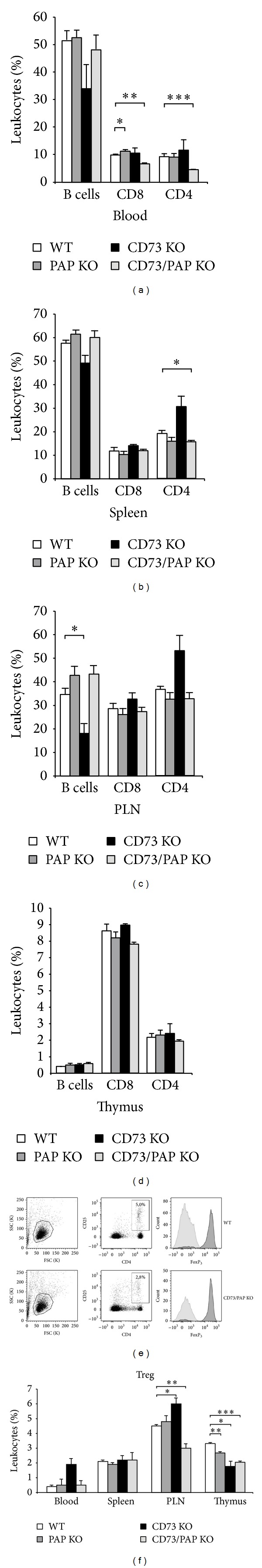
Synergistic effect of PAP and CD73 deficiency on the leukocyte subpopulations. Lymphocyte phenotypes in blood (a), spleen (b), lymph nodes (c), and thymus (d) of wild type mice (*n* = 7), CD73 single (*n* = 3), PAP single (*n* = 6), and CD73/PAP double knockouts (*n* = 4) (thymus: *n* = 3 for all genotypes) were analyzed using flow cytometry. (e) Regulatory T cells in lymph nodes. Examples of the FACS analyses of regulatory T cells in wild type and CD73/PAP KO mice are shown. In the left panels the gating strategy is shown and in the middle panels percentages of CD4^+^/CD25^+^ cells are indicated. Intensities of FoxP3 stainings of CD4^+^/CD25^+^ cells are shown as histograms in the right panels. Logarithmic fluorescence is on *x*-axis and the number of cells on *y*-axis. (f) Combined results of regulatory T cell analyses of different organs are shown as indicated. _ _*P ≤ 0.05, _ _**P ≤ 0.01, and _ _***P ≤ 0.001.

As there is no reliable antibody to analyze PAP expression at the protein level in mouse, we were mining the ImmGen consortium data bank that contains detailed analyses of different murine leukocyte and endothelial cell populations in respect to their gene expression profiles. PAP is expressed mainly in subsets of *γ* *δ* T cells and invariant natural killer T cells in thymus and both in CD4 and CD8 positive T cells in secondary lymphoid organs although generally in lower levels than CD73. A simplified comparison to CD73 expression is shown in Table 1. PAP deficiency alone did not change B cell, CD8, or CD4 populations in mice (Figures 5(a)–5(d)). The only statistically significant difference was seen in CD8^+^ cells in the blood, although in percentage-wise the increase is most likely biologically meaningless. The same held true when the lymphocyte populations were analyzed in blood and bone marrow of 8-month-old PAP mice (data not shown). We chose this time point as PAP knockout mice develop nonmetastatic prostate carcinoma at an old age [30]. The knockout prostates at this age contained more B cells than wild type prostates (6.6 ± 0.2% versus 1.4 ± 0.9%, *P* = 0.004). Also salivary glands contain more immune cells in these male mice, when they are young, but the difference disappears with aging [31]. The practically nonexisting alterations of major immune cell subpopulations in absence of PAP suggest that PAP does not seem to have any marked role in the immune system or its absence is compensated by CD73. Likewise, the changes in CD73 knockout mice were minor (Figures 5(a)–5(d)). Fewer B cells and slightly increased number of regulatory T cells were detected in peripheral lymph nodes.

### 3.4. CD73 and PAP Together Regulate the Number of CD4 and CD8 Cells in Blood and Number of Regulatory T Cells in Lymph Nodes and Thymus

In single knockouts, the number of regulatory T cells was significantly decreased in thymus and in the double knockouts both in PLN and thymus (Figures 5(e) and 5(f)). Still the intensity of FoxP3 expression, a marker for regulatory T cells, was at the same level in both double knockouts and wild type mice (Figure 5(e)). Thus, we may conclude that in thymus both CD73 and PAP contribute to number of regulatory T cells alone, but absence of both is needed to have an effect in PLN.

Although adenosine produced by CD73 on regulatory T cells propagates generation of more regulatory T cells via A_2A_ adenosine receptor [32], the number of regulatory T cells is very low and the main adenosine may come from peripheral lymph node stromal cells such as from endothelial cells. The stromal cells were not included in our enzymatic analyses showing nearly complete absence of ecto-5′-nucleotidase activity on isolated lymph node lymphocytes in CD73 KO mice.

Our finding of normal levels of regulatory T cells in organs other than in thymus, which had decreased number of regulatory T cells, and in lymph nodes, which had slightly increased number of regulatory T cells, is in agreement with the observation of Ehrentraut et al. [33], who did not find differences in the number of regulatory T cells between wild type and CD73 deficient mice in the lungs. This demonstrates that expression of CD73 overall is not needed for generation of regulatory T cells as such, although it is fundamental for the immunosuppressive function of these cells [9, 33].

The double knockout mice also have significantly lower percentages of CD8^+^ (32% reduction, *P* < 0.0001) and CD4^+^ cells (51% reduction, *P* < 0.006) in their blood and reduced number of CD4^+^ cells in the spleen (Figures 5(a) and 5(b)) further suggesting the synergistic effects of these two enzymes in controlling the lymphocyte pools.

**Figure 6:**
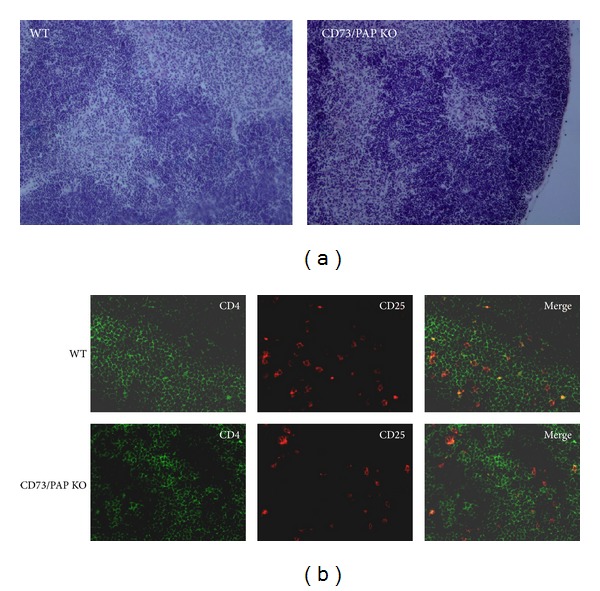
Thymuses of CD73/PAP double knockouts show comparable morphology and cell distribution to wild type mice. (a) Hematoxylin and eosin stainings of wild type and CD73/PAP knockout thymuses. (b) Immunofluorescence stainings of wild type and CD73/PAP knockout thymuses using anti-CD4 (green) and anti-CD25 (red) antibodies.

As thymus is the central organ in T cell development, we also analyzed thymic morphology and T cell subtypes of CD73, PAP, and double knockouts and compared them to those of wild type mice. The double knockout mice had normal thymic morphology (Figure 6(a)) and tissue location of different cell types was comparable between the genotypes. Double stainings of CD4 and CD25 are shown as examples in Figure 6(b).

Lymph nodes, spleen, and blood represent anatomically and functionally different environments for leukocytes providing unique cellular networks and extracellular milieu. This can also be realized by the differences in the expression of PAP and CD73 (Table 1). Therefore, our results showing organ-specific changes in lymphocyte subpopulations of double knockouts are understandable and emphasize the fact that, for example, in lymph nodes and spleen the factors promoting activation and differentiation of regulatory T cells are at least partially different and in spleen not markedly regulated by CD73 and PAP, although both enzymes are present in these organs. Regulatory T cells are produced in thymus and from there they disperse throughout the body to various organs in the hematolymphoid system or differentiated in the periphery. Regulatory T cells are heterogeneous regarding their phenotype, function, and epigenetic status and also the homing-associated molecules on regulatory T cells vary [34, 35]. This allows them to preferentially migrate to different organs such as peripheral lymph nodes, gut-associated lymphatic tissues, spleen, or nonlymphoid organs depending on the pattern of homing-receptors on their surface [35]. For example, CXCL12 highly expressed in bone marrow and splenic red pulp attracts regulatory T cells, while CCL25 recruits them to the small intestine. Thus, the regulatory T cells in different organs have phenotypically unique characteristics.

## 4. Conclusions

Generation and maintenance of the balance between different leukocyte subpopulations is a key element for proper functioning of the immune system in health and diseases. Anti-inflammatory adenosine produced by the AMPase activity of CD73 is central in immunosuppression. Although adenosine is important propagating regulatory T cells, lack of CD73 has surprisingly little effect on the number of regulatory T cells. This is the first report demonstrating the distribution of PAP in various mouse and human lymphoid tissues and its contribution, in conjunction with CD73, to the lymphoid purine homeostasis and the balance of leukocyte subpopulations in various organs. However, alone absence of PAP does not have any significant effect outside the thymus, but, combined to the deficiency with CD73, the consequences become significant. Further work is needed to elucidate the exact mechanism of the mode of action of PAP in the immune system.

## Figures and Tables

**Table 1:** Expression profiles of CD73 and PAP at the mRNA level in different lymphoid tissues∗.

	CD73 (*Nt5e*)	PAP (*Acpp*)
Thymus	*γδ* T cells∗∗	*γδ* T cells∗∗
	CD4 single positive	iNKT precursors∗∗
	Double negative thymocytes	
	CD8 single positive, mature	
	iNKT∗∗∗ precursors∗∗	

Lymph nodes	CD4 memory	CD8 naïve
	CD8 naïve	
	CD8 memory	
	Treg	
	Blood endothelial cells	

Spleen	Germinal center B cells	CD4 naïve
	Memory B cells	CD4 memory
	*γδ* T cells∗∗	*γδ* T cells∗∗
	CD4 naïve	CD8 naïve
	CD4 memory	CD8 memory
	CD8 naïve	iNKT cells
	CD8 memory	
	Treg	
	iNKT cells	
	NK cells∗∗	

∗The data has been collected from the Immunological Genome Project (http://www.immgen.org/databrowser). The cell types with an expression value (EV) >120 for a given gene are listed (95% confidence of positive expression).

∗∗Only in selected subpopulations.

∗∗∗iNKT = invariant natural killer T cells.
